# The triarchy of L2 learners’ emotion, cognition, and language performance: Anxiety, self-efficacy, and speaking skill in lights of the emerging theories in SLA

**DOI:** 10.3389/fpsyg.2022.1002492

**Published:** 2022-09-20

**Authors:** Yuxia Ma

**Affiliations:** College of Foreign Languages and Cultures, Chengdu University, Chengdu, China

**Keywords:** foreign language anxiety, self-efficacy, speaking skill, oral communication, emotion, cognition

## Abstract

Given the bond between emotion and cognition underlying the broaden-and-build theory of positive emotions, positive and negative emotions have critical roles in cognitive skills. The aim of this review was to probe into the triarchic relationship between L2 learners’ cognition, emotion, and language performance, reflected in the bond between self-efficacy, foreign language anxiety, and speaking skill, in light of the main emerging theories in the field of SLA underpinning this relationship. Moreover, the theoretical foundations, such as learners’ cognitive performances, Bandura’s self-efficacy theory, control-value theory, and positive psychology theory, were explicated in order to justify the relationship between self-efficacy and oral communication skill. Furthermore, the underlying theories such as attentional control theory, complex dynamic system theory, affective filter theory, and cultural schema theory, which relate foreign language anxiety to speaking skill were scrutinized in this review. The review also expounded on the implications and future directions for EFL teachers, material designers, teacher educators, educational policy-makers, and advisors. The ideas can improve their awareness of learner self-efficacy, foreign language anxiety, and oral communication skill in educational contexts.

## Introduction

Mastery of speaking skill in English is a priority for many second-language and foreign-language learners. The reason lies in the fact that speaking, as a significant skill, is the prime means of communication (Sadullaevna and Safarovna, 2020). Likewise, speaking in a foreign language will boost practical communication skills in a way that reading or listening alone will never do, which is why speaking is stressed so much among L2 language learners (Al Zoubi, 2018). Many language learners find it difficult to express themselves in spoken language. They often have difficulty expressing their thoughts effectively. They stop talking because they face psychological barriers or can’t find the right words and phrases. If teachers want to help learners dominate problems in learning the speaking skill, they need to identify some factors that affect their speaking performance. Speaking performance of learners is influenced by factors such as environments, affective factors, listening skill, and feedback during speaking tasks (Tuan and Mai, 2015). Learners of English as a second language (ESL) often encounter discouraging situations, in and out of the classroom, that make it difficult for them to maintain the positive cognitive state needed to achieve their desired learning objectives. In order for students to successfully face such challenges, cognitive interventions may be needed to counteract the negative cognitive state that students might experience (Bandura, 2006). One such state is a lack of self-efficacy. Self-efficacy refers to an individual’s belief about his or her capabilities to perform the specific tasks required to produce certain outcomes (Bandura, 2012).

According to Bandura (2001 p. 10), “unless people believe [that] they… by their actions… can produce desired results… they have little incentive to act or to preserve in the face of difficulties”. There is a growing body of self-efficacy research in educational contexts among teachers (Seifalain and Derakhshan, 2018; Fathi and Derakhshan, 2019; Fathi et al., 2020a,^2021^), and learners (Ghonsooly et al., 2012; Elahi Shirvan et al., 2018; Fathi and Derakhshan, 2018; Fathi et al., 2020b). Moving on to the important individual-difference variable of this review, anxiety is another most influential factor in the domain of L2 learning (Kasbi and Elahi Shirvan, 2017; Elahi Shirvan and Taherian, 2018; Elahi Shirvan et al., 2018; Fathi et al., 2020b; Saghafi and Elahi Shirvan, 2020). In the presence of anxiety, L2 knowledge often gets affected and deteriorates (Dörnyei, 2005). According to Arnold and Brown (1999), “anxiety is quite possibly the affective factor that most pervasively obstructs the learning process” (p. 8). Based on Dornyei and Stephen (2015), anxiety is not a monolithic factor, but rather a complex construct that is made up of different components.

Yashima et al. (2004) strongly suggested that future research on oral communication in English should focus on contextual or situational positive and negative variables that make a person enhance oral communication in English classroom settings, and outside classes. In other words, it is necessary to look into how negative and positive emotional constructs influence communication behavior. Accordingly, this review examines the theoretical underpinnings of the relationship between self-efficacy, foreign language anxiety and speaking skill. The results of this study will also influence language teachers, teacher trainers, and course/program material designers. This research can enable English teachers to understand what variables determine oral communication in English among EFL learners and allow them to design effective teaching pedagogy and activities to improve oral skill.

Given the advent of the positive psychology movement in the field of SLA in recent years (Wang et al., 2021), attention has been drawn to the integration of both affective and cognitive variables and its association with language-related outcomes such as oral communication. The broaden-and-build theory of positive emotions (Fredrickson, 2001) puts emphasis on the tight link between language learners’ affective and cognitive traits. Thus, language learners’ belief systems and appraisal of their linguistic competencies are supposed to be susceptible to their positive emotions. Despite the salience of this theoretical framework of positive emotions, the link between cognitive and emotional variables and their ties with L2 outcomes have hardly been addressed yet.

## Review of literature

From a theoretical perspective, it should be noted that the link between L2 learners’ emotion and cognition, as reflected in anxiety and self-efficacy, can be supported by Fredrickson’s broaden-and-build theory of positive emotions (Fredrickson, 2001). Based on this theory, individual cognitive capacity and belief systems can be built and broadened by their positive emotional experience. In what follows, since speaking skill (oral communication) has been mainly investigated as an outcome of anxiety and self-efficacy in the field of SLA, and also due to the word limits of the journal, it has been incorporated in the sections on its association with self-efficacy and anxiety.

### The concept of learner self-efficacy

The notion of self-efficacy is summarized by Oxford and Shearin (1994), as a “broadened view of expectancy which is drawn from social cognition theory” (p. 21). According to Bandura (1986), self-efficacy refers to “people’s judgments of their capabilities to organize and execute courses of action required to attain designated types of performances” (p. 391). He asserted that self-efficacious individuals rely on their own competence to deal with demanding activities, and carry out the required strategies to be effective in forthcoming situations. Jeong et al. (2021) stated that self-efficacy specifies students’ confidence in arranging their learning process and influences their apprehension of cognitive growth. Schunk and Pajares (2010) also indicated that individuals with higher levels of self-efficacy are inclined to have higher intrinsic interest, set themselves thought-provoking objectives, and keep a strong commitment to activities.

Bandura (1997) listed four primary sources of self-efficacy beliefs as (1) enactive mastery experiences, (2) vicarious experiences, (3) verbal persuasion, and (4) the physiological and affective state of an individual. Zhang and Ardasheva (2019) stated that enactive mastery experiences, are the most significant cause of self-efficacy. They mentioned that enactive mastery experiences are related to an individual’s insight over his/her own capability to positively undertake a specific task informed by earlier accomplishments. They mentioned that enactive mastery experiences are related not only to individuals’ perception of their capability, but also to the task’s difficulty, and the amount of effort they will exert to accomplish the task. According to Wilde and Hsu (2019), vicarious experiences, as the second source of self-efficacy, are concerned with the social comparison of a person’s performance to that of others with similar abilities. El-Abd and Chaaban (2021) asserted that observing others’ comparable capabilities can improve one’s self-efficacy by approving the sufficiency of his/her own knowledge, abilities, and approaches. Verbal persuasion, the third source of self-efficacy, refers to “socially persuasive feedback, comments by significant others regarding one’s performance” (Bandura, 1997, p. 20). Wangwongwiroj and Yasri (2021) mentioned that constructive comments emphasizing an individual’s aptitudes or achievements would improve self-efficacy. The physiological and affective state of an individual, the fourth source of self-efficacy, is related to individuals’ capability to control bodily and emotional stress reactions (e.g., breathing and anxiety) over task performance (Webb-Williams, 2018).

### Underpinning theories in the relationship between learners’ self-efficacy and oral communication

Bandura’s self-efficacy theory is considered a theoretical construct in the relationship between self-efficacy and oral communication. According to the theory of self-efficacy (Bandura, 1997), individual behavior is subject to awareness of and congruence with the anticipated results, which inevitably affect a person’s learning outcomes. Nur and Butarbutar (2022) stated that Bandura’s self-efficacy theory stems from motivation. In simple terms, motivation is about the students’ self-view of their ability to complete tasks. When operating at that own rate, it could be suggested that the improvement in self-awareness could improve self-efficacy as well. Based on this theory, self-efficacy has an impact on people’s feelings, beliefs, and behaviors. In terms of emotions, a low sense of self-efficacy is associated with despair, anxiety, and helplessness. Individuals with low self-esteem and pessimistic perceptions of their accomplishments and personal growth are equally vulnerable. In terms of thinking, a high sense of competence aids cognitive processes and scholastic accomplishment (Nur and Butarbutar, 2022). People with strong self-efficacy put in more effort and remain with it long after taking action than those with low self-efficacy. When they experience impediments, they improve faster and remain committed to their purposes (Habibi and Yazdani, 2016). Using Bandura’s self-efficacy theory, Zhang et al. (2020) studied English for academic purposes (EAP) and English-as-a-foreign-language (EFL) literature, found a significant relationship between self-efficacy and speaking performance. Zhang and Ardasheva (2019) examined the relationship between four sources of Chinese English learners’ self-efficacy, including enactive mastery experience, vicarious experience, verbal persuasion, physiological, affective states, and their oral communication. Their findings largely support Bandura’s hypothesized sources of self-efficacy, with enactive mastery experience, vicarious experience, verbal persuasion, but not physiological affective states, significantly predicting learners’ oral communication skill.

Self-efficacy, as a positive emotional state, can influence foreign language performance. Based on Pekrun’s (2006) control-value theory, emotional states influence learners’ motivation to learn, their learning strategies, and self-regulated learning, thereby influencing their learning achievement. Pekrun et al. (2002) stated that the control-value theory postulates that the subjective control and subjective value, as the types of cognitive appraisals, are central components of achievement emotions.

Luo et al. (2016) defined subjective control as “an individual’s perceived causal influence of the self over achievement activities and outcomes” (p. 2). They mentioned that subjective control can take the forms of retrospective causal attribution and prospective expectancy of success or failure, often operationalized as self-efficacy, academic self-concept, or academic control. There are two types of subjective value. Intrinsic values stem from academic studying, including a feeling of pleasure or satisfaction from doing academic tasks or achieving academic success. Extrinsic values are also described as individual’s supposed instrumental usefulness of academic actions or outcomes for achieving other goals. These two types of appraisals combine to evoke various achievement emotions. Pekrun (2017) stated that control-value theory is considered an inclusive framework for examining antecedents and consequences of student emotions. Roick and Ringeisen (2017) argued that the control-value theory offers a sequence of cognitive predictors assumed to trigger anxiety in performance-related test situations. They mentioned that high dispositional control beliefs like self-efficacy improve mastery perceptions in achievement situations, thus reducing the anticipated risk of failure.

Positive psychology is regarded as another theory that can relate learner self-efficacy to linguistic skills. Traditionally, psychologists have highlighted the deficiencies and negative emotions among learners and teachers, and they have made an effort to decrease them (Deweale and Alfawzan, 2018; Derakhshan et al., 2021). Positive psychology, as a modern approach to learning of foreign language, has been expanded in recent years (Wang et al., 2021). It tries to illuminate the optimal educational situations and processes for the achievement of learners and teachers (Jiang, 2020). Fredrickson and Cohn (2008) stated that positive affect is the major trigger of active engagement with the learners’ environment and his/her will to participate in classroom activities. Fathi et al. (2021) categorized the constructs of positive psychology into empathy, enjoyment, happiness, contentment, optimism, tolerance, flow, love, and mindfulness, which can result in a person’s satisfaction, self-efficacy, and success. Through applying principles of positive psychology, higher education practitioners can help improve self- esteem, self-concept, and self-efficacy among college learners (Costello and Stone, 2012). On the other hand, Abdolrezapour (2018) stated that positive emotions tend to widen learners’ viewpoint and open their viewpoint to absorb the language. Also, MacIntyre et al. (2019) asserted that positive psychological interventional studies used numerous approaches in order to enhance EFL learners’ skills.

### The concept of foreign language anxiety

Anxiety, as a negative emotion, is described anxiety as a state of sensitive awareness correlated with an augmentation in stress as a result of ambiguity (Wells and Matthews, 1996). Many researchers (e.g., Aydin, 2018; Ulupinar, 2018; Russell, 2020; Hu et al., 2021; Toyama and Yamazaki, 2021) have highlighted foreign language anxiety in the field of education. In an educational context, Horwitz et al. (1986) defined foreign language anxiety as “a distinct complex of self-perceptions, feelings and behaviors related to classroom language learning arising from the uniqueness of the language learning process” (p. 127). Gardner and MacIntyre (1993) pointed out that “foreign language anxiety is fear or apprehension occurring when a learner is expected to perform in the second or foreign language” (p. 59). They linked it to the stimulation of the autonomic nervous system.

Horwitz et al. (1986) categorized foreign language anxiety construct into (a) test anxiety, (b) fear of negative evaluation, and (c) communication apprehension. Cakici (2016) mentioned that test anxiety is regarded as learners’ fear of experiencing failure in academic evaluation in testing contexts. He also stated that negative evaluation is concerned with worrying about individuals’ negative judgments. Communication apprehension, based on Horwitz et al.’s (1986) definition, refers to “a distinct complex of self-perceptions, beliefs, feelings, and behavior related to classroom language learning arising from the uniqueness of the language-learning process” (p. 28). Communication apprehension is a critical element in limiting the received comprehensible input, and it plays a vital role in determining accomplishment in educational contexts (Darmawangsa et al., 2020).

Studies have shown several sources of foreign language anxiety, such as minor levels of self-confidence (Tridinanti, 2018), lower levels of self-efficacy (Bensalem, 2018), low levels of grit (Liu and Wang, 2021), lack of practice (Bárkányi, 2018), low levels of language proficiency (Teimouri et al., 2019), low levels of emotional intelligence (Chen et al., 2021), fear of making mistakes (Suparlan, 2021), insufficient input flooding, first language overuse, cultural background factors (Shan et al., 2020), socio-economic status (Ali et al., 2021), and teachers’ negative impression about learners’ academic performance (Liu and Wu, 2021).

### Underpinning theories in the relationship between learners’ foreign language anxiety and oral communication

To increase students’ desire to communicate and use the language, affective factors should be carefully considered (Lee and Lee, 2020). Learners’ cognitive performances have been affected by their foreign language anxiety. Mede and Karaırmak (2017) pointed out that “foreign language anxiety significantly affects the domains of language achievement, learners’ actual proficiency and performance, gender, prior foreign language experience, negative evaluation, and self-evaluation” (p. 119). Foreign language anxiety has been widely considered a leading factor in academic achievement and language proficiency (MacIntyre, 2017). Studies have testified that higher levels of anxiety are negatively correlated with foreign language proficiency, and with positive orientation, and peer emotional support (Zheng and Cheng, 2018). Regarding the relationship between foreign language anxiety as a negative type of emotion and cognitive skills, Fallah and Movahed (2014) indicated that there was a negative correlation between foreign language anxiety, and listening, reading, speaking, and writing skills, and learners’ academic achievements were negatively influenced by anxiety. Hu et al. (2021) also found that learners’ foreign language anxiety was negatively associated with their foreign language skills. Aghajani and Amanzadeh (2017) investigated the relationship between foreign language anxiety and oral communication performance, and they tried to determine to what extent foreign language anxiety affects the oral communication performance among adult EFL learners. They concluded that there is a strong negative correlation between the two variables which acknowledged the negative role of high anxiety in oral communicative performance of students.

The negative relationship between foreign language anxiety and oral performance can be attributed to Eysenck et al.’s (2007) attentional control theory. Muris et al. (2007) stated that attentional control theory explains the regulation of different attentional processes within the attentional system. They mentioned that attentional control refers to the individuals ‘capability to flexibly concentrate and change their attention based on existing objectives. Wells and Matthews (1996) mentioned that anxiety can be characterized by inadequate control over disturbing opinions and attentional and cognitive predispositions, leading to an excessive concentration on undesirable incentives. It is worth noting that anxiety is negatively affected by input, processing and output as the phases of cognitive processing (MacIntyre and Gardner, 1994). Eysenck et al. (2007) argued that “attentional control theory proposes that increased anxiety results in reduced attentional control such that the effect of the goal-directed system is reduced and the influence of the stimulus-driven system is increased through preferential processing of task-irrelevant threat related stimuli” (p. 30). Eysenck and Derakshan (2011) asserted that attentional control theory also suggests that poor attentional control can be a risk factor for the development of high trait anxiety. They mentioned that threatening stimuli would capture attention, and the more state or trait anxiety individual experiences, the more likely ambiguous information would be interpreted in a threatening way and hence capture attention. Zhou et al. (2020) mentioned that Eysenck et al.’s (2007) attentional control theory can justify the reason behind the negative correlation between foreign language anxiety and cognitive processing performance. They maintained that foreign language anxiety limits the attentional control of learners in adverse situations, so that learners are more prone to threat-related stimuli or distractors.

The complex dynamic system theory is also used to justify the relationship between anxiety and oral communication. On the issue of second language speaking and anxiety from an ecological perspective, Kasbi and Elahi Shirvan (2017) conducted a case study on four intermediate level female students with an average age of 15 at five sessions of 90 min, to investigate EFL learners’ anxiety in oral performance from an ecological perspective based on nested ecosystems model and complex dynamic system theory. Their findings indicated that the events within the dynamics of classroom ecology can influence EFL learners’ anxiety in speaking performance differently. Highly anxious students can be very relaxed even in situations where others with low anxiety are highly anxious. They concluded that in some situations, context was important and unexpected anxiety increased in some students. On the other hand, in some cases “powerful forces like oral informal class assessments, teachers’ questions, did not have any impact on all the participants in a similar way, which was in line with the complex dynamic system theory principles of non-linearity in system behavior” (p. 18).

Another theory that can justify the relationship between foreign language anxiety and oral communication is affective filter theory. Wang and Wu (2020) indicated that when learners’ affective filters are high, the mental block created makes it impossible for them to acquire or be comfortable with the input. Thus, that filter level will create a hostile environment in which the students feel insecure about themselves and their capacities to use the language to communicate. Garcia Uquillas (2021), using affective filter theory, found out that anxious learners underperform in foreign language production he argued that affective filter, including emotional constructs of anxiety, motivation, and self-efficacy, influences foreign language learning. He mentioned that instructors should lower the affective filter because all of the factors involved in it, such as higher anxiety, lower motivation, and lower self-efficacy may bring a negative influence on language production. That is, the affective filter plays an important role in the foreign language learning process. All the aspects involved in it do affect the way in which learners acquire the language. Therefore, educators should have the capability to preserve the filter on the right levels. Fernández Silva (2019) investigated the effect of using audio-visuals on EFL learners’ affective filter to enhance their oral communication. His study demonstrated that audio-visual materials can lower learners affective filter, which reduces their foreign language anxiety and their speaking fluency increases. His study revealed that some different language activities can facilitate the reduction of negative emotions like foreign language anxiety, which, in turn, boosts learners’ communicative skills. He concluded that as students are motivated, with low levels of anxiety and with confidence, they can make use of more vocabulary, grammar, etc., to finally become more fluent when they speak. Nath et al. (2017) asserted that some learners had a high filter which had negative consequences on their oral production of the target language, whereas other learners had a low filter that encouraged the appropriate oral production of the language and use of the metacognitive strategies for their own sake. In a few words, the affective filter has an incidence on how much students can make use of strategies to overcome difficulties when learning a language.

Another theory for justifying the relationship between foreign language anxiety and speaking skill is Anderson’s (1977) cultural schema theory. Turkan and Çelik (2007) argued that the consideration of cultural issues is very significant in foreign language learning. They asserted that familiarizing with cultural issues can reduce anxiety, and it can be effective in foreign language communication. Using Anderson’s cultural schema theory, Diep et al. (2022) found a significant correlation between Indonesian EFL learners’ foreign language anxiety and oral communicative skill. They found out that cultural-based instruction can be a mediating factor in developing oral performance and reducing foreign language anxiety. Based on learners’ cultural schema theory, they argued that cultural familiarity can help learners outperform in foreign language learning. They also mentioned that triggering learners’ cultural schema can develop speaking skill. Bilokcuoglu (2014) stated that triggering background knowledge can result in language learning achievement. He asserted that learners’ anxiety in foreign language learning can be reduced by schemata activation. This type of theory is based on preceding knowledge about the educational context, which can contribute learners to link new information to that earlier knowledge. This association helps them in language learning education (Bilokcuoglu, 2014).

## Implications and suggestions for further research

This review examined the theoretical underpinnings of the relationship between EFL learners’ self-efficacy and oral communication. Some theories, such as Bandura’s self-efficacy, Pekrun’s (2006) control-value, and positive psychology have been presented to elucidate the relationship between EFL learners’ self-efficacy and oral communication. Also, this review considered the theoretical foundations of foreign language anxiety and oral communication. The theories, such as attentional control theory, complex dynamic system, affective filter, and cultural schema can be the basis for the relationship between foreign language anxiety and oral communication. Revisiting the associations among the affective, cognitive, and performance-related variables within the L2 context contributes to our achievement of a deeper understanding of the credibility of the broaden-and-build theory of positive emotions in this context. This means that language learners’ main sources of self-efficacy such as mastery and vicarious experiences as well as the psychological feedback and states should be viewed from the perspective of the emotions they experience. Any mastery or vicarious experiences *per se* might not lead to high levels of L2 skills such as oral skills unless learners go through positive emotions such as enjoyment. This review includes some pedagogical implications for teachers, learners, syllabus designers, teacher educators, educational policy-makers, and advisors. Also, different associations and related authorities can benefit from this review. For instance, a better understanding of emotional and cognitive factors in oral communication in the target language may help language teachers improve the communicative language teaching approach and curriculum design to provide more communication opportunities for language learners, more importantly, encourage actual engagement in communication behaviors, and finally, facilitate second/foreign language learning and acquisition. More specifically, language instructors can enhance the level of oral production through the following ways: raising students’ opportunity to talk by reducing the amount of teacher talk and allowing adequate wait-time; letting students produce language without restrictions, uncontrolled use of language; take responsibility to engage all students evenly and equally in classroom activities; videotaping themselves in the classroom, reflect on their interactional behavior to see if it has extended or limited the opportunity for the students to enter dialogs; increasing their own awareness of what interaction strategies work or do not work with specific students, and giving the instruction that lends itself to more giving and receiving of unpredictable information.

Another implication of this review is to develop learners’ self-efficacy to enhance their oral communication. Teachers may also benefit from this review in that they must be aware of the positive correlation between the learners’ self-efficacy and their proficiency. They have to take care of and help the learners who suffer from poor self-efficacy and help them improve in terms of self-regulation, self-esteem, and self-concept as these traits form the bases of self-efficacy (Ghonsooly and Elahi, 2010). It is believed that instructing learners on techniques to improve their self-efficacy should be given the same priority as other language skills in the EFL context. Self-efficacy could have an important role in the application and use of the approaches and methodologies in the EFL context. Instructors can use moderately-difficult activities which can empower learners with low levels of self-efficacy. The activities should not be too difficult to curb learner self-confidence in doing tasks. Teacher support, including, scaffolding, assigning sufficient time, decomposing complex tasks into simple phases, and explicating the task in technology-supported education is influential for the enhancement of learner self-efficacy. This can produce insight into the reasonable challenge and equalizes the complexity of technology-supported tasks. Praising and giving feedback to learners are also important for the improvement of learner self-efficacy. Moreover, teachers should not compare the performances of learners with each other. Teachers can provide learners with some strategies such as self-verbalization. For example, they can motivate learners to express the procedure of learning grammatical points or vocabulary aloud and give feedback on their effort. Moreover, teachers can set a cooperative context, rather than a competitive one, to increase learner interaction and scaffolding, improving learner self-efficacy. They can also ask learners to write comments about their feelings and progressions in technology-supported contexts (see Brown, 1994; Aghaei et al., 2020).

The review revealed that anxiety has a detrimental effect on learners’ speaking performance. Thus, in order to encourage students’ oral communication and enhance their involvement in the class activities, teachers need to reduce students’ FL anxiety in the class by making the class atmosphere more friendly and comfortable. In a less threatening atmosphere, learners’ anxiety may reduce while their level of perceived competence increase. Therefore, teachers need to make a relaxed and intimate atmosphere to increase oral communication skill among students and as a result, enhance language learning in the class. Using activities like role play, small group work, pair work, small group discussion, and presenting the lecture may make the class atmosphere less threatening for students. Moreover, teachers should not directly suppose that poor performance by unresponsive and unwilling students is absolutely due to their lack of motivation, aptitude or ability; they should instead identify the possibility that some students might be suffering from anxiety. Hence, rather than disregarding students’ speaking anxiety or leaving them to cope with it on their own, EFL teachers should consider it as a reality in EFL classrooms and attempt to prepare learners with useful strategies to help them cope with multiple anxiety-producing situations. Similarly, it is suggested that EFL teachers hold seminars and workshops to inform students about the nature of speaking anxiety and how it can be effectively handled. It is proposed that EFL teachers provide English classes with useful strategies such as communication games. Teachers, via applying such games, may keep students from emotional stress through effective communication, help them build habits of interaction and participation, assist them in constructing their sense of enjoyment, enthusiasm, and feeling of satisfaction, enhance their predisposition and motivation toward language learning, work together in a friendly relationship, confidential, relaxed, supportive and sociable atmosphere, and consequently to foster their self-confidence to be able to overcome their own anxiety. It is also recommended that teachers while communicating with their classes, comprehend learners’ concerns in their learning process, and try to open the classroom climate.

To support EFL learners to become better communicators, it seems to be important to handle communication challenges, specifically in speaking area. To this end, it is suggested that textbook compilers, before designing a specific textbook, firstly, attempt to recognize the extensive area of sources of speaking anxiety in EFL classrooms, to obtain a comprehensive and realistic view from students’ speaking difficulties, then include effective and useful activities in English textbooks to help teachers reduce learners’ communication apprehension and assist students to cope with multiple speaking anxiety-generating situations. To this aim, textbook compilers are recommended to design or compile applicative communication games and integrate them precisely into lessons. These games-oriented lessons may help teachers be able to construct a friendly relationships amongst students, establish a supportive and stress-free classroom atmosphere, promote learners’ motivation toward language learning, and accordingly reduce the level of speaking anxiety in EFL learners.

Moreover, teacher training courses can reach their ideal goals by considering the importance of self-efficacy, anxiety, and willingness to communicate. In order to increase learner self-efficacy, they can implement some instructional changes in a large population of teachers by holding academic workshops. Educational policy-makers should hire experienced teachers, as the instructive experience can be an important issue for increasing self-efficacy and lowering anxiety among learners. They can ask teachers to do their best within varied educational contexts. They must build up teaching effectiveness through providing contexts for observations of other teachers’ activities and mastery experiences to decrease foreign language anxiety in particular ranges of the instruction. They should also provide critical thinking, creativeness, and motivation to the education in classrooms, which encourages self-efficacy (see Aghaei and Rad, 2018). The importance of self-efficacy, anxiety and speaking skill can motivate advisors to expand their horizons to identify learners’ sources of foreign language anxiety, and self-efficacy, and to probe the reasons for increasing oral communication skill.

Many cross-sectional studies have been done on the role of emotional constructs in learners’ oral communication. A longitudinal qualitative study is necessary to examine the effect of emotional constructs on learners’ oral communication in English in various situations both inside and outside class. It would also prove fruitful to examine instructor’s perceptions of the communication behaviors of their learners. In order to increase learners’ speaking skill, teachers need to monitor various aspects of teaching styles and classroom management. Moreover, the relationship between oral communication skill and other positive psychological constructs, such as academic engagement, well-being, enjoyment, and resilience can be investigated in the future (Rajabi and Ghezelsefloo, 2020). Factors that could be more closely examined include the relationship between age, gender, background, personality, intercultural communication experiences, and learners’ oral communication skill. Finally, the relationship between positive emotional constructs and writing, reading, and listening skills should be studied in the future.

## Author contributions

The author confirms being the sole contributor of this work and has approved it for publication.
